# Posterior concentration of *Wolbachia* during the early embryogenesis of the host dynamically shapes the tissue tropism of *Wolbachia* in host *Trichogramma* wasps

**DOI:** 10.3389/fcimb.2023.1198428

**Published:** 2023-06-22

**Authors:** Jin-Cheng Zhou, Qian-Jin Dong, Dan Shang, Su-Fang Ning, Huan-Huan Zhang, Ying Wang, Wu-Nan Che, Hui Dong, Li-Sheng Zhang

**Affiliations:** ^1^ College of Plant Protection, Shenyang Agricultural University, Shenyang, China; ^2^ State Key Laboratory for Biology of Plant Diseases and Insect Pests, Institute of Plant Protection, Chinese Academy of Agricultural Sciences, Beijing, China; ^3^ Institute of Vegetable, Tibet Academy of Agriculture and Animal Husbandry Sciences, Lhasa, China

**Keywords:** endosymbiont, thelytokous parthenogenesis, vertical transmission, biological control, *Trichogramma dendrolimi*, *Trichogramma pretiosum*, egg parasitoid

## Abstract

**Introduction:**

The bacterial endosymbiont, *Wolbachia* spp. induce thelytokous parthenogenesis in certain parasitoid wasps, such as the egg parasitoid wasps *Trichogramma* spp. To complete the cycle of vertical transmission, *Wolbachia* displays efficient transovarial transmission by targeting the reproductive tissues and often exhibits strong tissue-specific tropism in their host.

**Method:**

The present study aimed to describe the basic *Wolbachia* distribution patterns that occur during the development of *Wolbachia*-infected, thelytokous *Trichogramma dendrolimi*, and *T. pretiosum*. We used fluorescence *in situ* hybridization (FISH) to investigate *Wolbachia* signal dynamics during early embryogenesis (from 30 to 120 min). *Wolbachia* titers and distributions from the embryo to adult stages of *Trichogramma* after early embryogenesis were detected by absolute quantitative polymerase chain reaction (AQ-PCR) and FISH. The symmetry ratios (SR) of the *Wolbachia* signals were calculated using the SR odds ratios in the anterior and posterior parts of the host. The SR was determined to describe *Wolbachia* tropism during early embryogenesis and various developmental stages of *Trichogramma*.

**Results:**

*Wolbachia* was concentrated in the posterior part of the embryo during early embryogenesis and the various developmental stages of both *T. dendrolimi* and *T. pretiosum*. *Wolbachia* density increased with the number of nuclei and the initial mitotic division frequency during early embryogenesis. The total *Wolbachia* titer increased with postembryogenesis development in both *T. dendrolimi* and *T. pretiosum*. However, the *Wolbachia* densities relative to body size were significantly lower at the adult and pupal stages than they were at the embryonic stage.

**Discussion:**

The present work revealed that posterior *Wolbachia* concentration during early host embryogenesis determined *Wolbachia* localization in adult wasps. By this mechanism, *Wolbachia* exhibits efficient vertical transmission across generations by depositing only female *Wolbachia*-infected offspring. The results of this study describe the dynamics of *Wolbachia* during the development of their *Trichogramma* host. The findings of this investigation helped clarify *Wolbachia* tropism in *Trichogramma* wasps.

## Introduction

The bacterial endosymbiont, *Wolbachia* spp. resides in the cells of more than 40% of all terrestrial arthropod species (LePage et al., 2017; Salje, 2021). To ensure their transovarial transmission from host mothers to offspring, *Wolbachia* must invade host reproductive tissues and often cause reproductive alterations in their hosts such as cytoplasmic incompatibility, parthenogenesis, male killing, and feminization (Kaur et al., 2021). *Wolbachia* can induce thelytokous parthenogenesis in certain parasitoid wasps, such as *Trichogramma* spp. (Huigens et al., 2000; Zhou et al., 2022a; Zhou et al., 2023). Thelytokous parthenogenesis, also known as female-producing parthenogenesis, is defined as the production of female offspring without genetic contribution from paternal males (Ma and Schwander, 2017). In host wasps, *Wolbachia* can convert male-destined haploid eggs into homozygous diploid female eggs by inducing gamete duplication during the initial mitotic division (Wu et al., 2020; Zhou et al., 2022b).

The penetrance of parthenogenesis in *Trichogramma* wasps relies on the parthenogenesis-inducing (PI) *Wolbachia* titers in their germ line (Zhou et al., 2022b). *Wolbachia* invade host eggs, and complete vertical transmission cycles by targeting the germ line during host development, and exhibit strong tissue-specific tropism (Pietri et al., 2016). This tropism is determined by three different strategies (Landmann et al., 2012). First, the concentration of *Wolbachia* in the germ line is determined by their posterior localization in the early embryo, which is influenced by both the symmetric and asymmetric cell division segregation patterns during oogenesis and early embryogenesis (Albertson et al., 2009; Landmann et al., 2010). Second, this tropism is associated with cell-to-cell transmission (Serbus et al., 2008; Newton et al., 2015; Pietri et al., 2016). A previous study examined the penetration of *Wolbachia* into host cells and their subsequent migration from somatic tissues to the germ line, elucidating this tropism (Guo et al., 2019). Third, *Wolbachia* activates immune responses and modifies their titers in different tissues (Masson et al., 2016). As a rule, the host germ line originates from the posterior pole of the egg and the early embryo (Pietri et al., 2016). The fundamental distribution pattern of *Wolbachia* in developing host adult tissues may be determined by their asymmetrical distribution in the early embryo. Several studies have proposed that the tissue-specific distributions of *Wolbachia* are influenced by the localization pattern of these endosymbionts in the host embryo.

The distributions of *Wolbachia* during embryogenesis have been documented in numerous host species, revealing different patterns. Previous studies have reported that *Wolbachia* may exhibit an anterior concentration (Wright & Barr, 1981; Boyle et al., 1993; Guo et al., 2019), a posterior concentration (Zchori-Fein et al., 1998; Strunov et al., 2022), or a broad distribution (Breeuwer & Werren, 1990; Pintureau et al., 2000; Strunov et al., 2022) in different *Wolbachia*-host systems. Despite documentation of *Wolbachia* distribution in parasitoid embryos of *Nasonia* and *Trichogramma* wasps, different results have been reported even among closely related parasitoid species. For instance, the *Wolbachia* wVitA strain localizes to the posterior end at low density in *N. vitripennis* embryos, while in *N. giraulti* embryos, it localizes to the posterior end at high density and then spreads toward the anterior end (Chafee et al., 2011; Funkhouser-Jones et al., 2018). *Wolbachia* has been reported in the posterior poles of *T. cordubensis* embryos, whereas the posterior concentration of *Wolbachia* is not found in *T. dendrolimi* (Pintureau et al., 2000). However, the study on *Trichogramma* embryos was conducted at only one-time point. Little is known about how the dynamic distribution of *Wolbachia* during early embryogenesis shapes the germ-line-specific distribution of *Wolbachia* in adult *Trichogramma*. Further comprehensive studies are required to elucidate this process.

The egg parasitoid wasps *Trichogramma dendrolimi* Matsumura and *T. pretiosum* Riley have been widely used in agriculture and forestry for the biological control of various lepidopteran pests (Lindsey et al., 2018; Zhou et al., 2019). The sex determination mechanism of *Trichogramma* wasps is haplodiploid, where females arise from a diploid fertilized egg and males develop from unfertilized haploid eggs. *Wolbachia* infection often induces thelytokous parthenogenesis in *Trichogramma* wasps by triggering gamete duplication in early embryos. At least 15 *Trichogramma* species have been documented to exhibit thelytokous parthenogenesis when infected with PI-*Wolbachia* (Ma and Schwander, 2017; Wu et al., 2020; Zhou et al., 2022b). Compared to uninfected bisexual parasitoid wasps, *Wolbachia*-infected thelytokous wasps produce nearly 100% female offspring, which are effective in controlling insect pests (Zhou et al., 2020). Our previous study revealed that the potency of parthenogenesis in *Wolbachia*-infected wasps is related to the *Wolbachia* titers in the germ line. This study aimed to elucidate the dynamics of *Wolbachia* during early embryogenesis in the host and establish how these dynamics shape tissue-specific distributions of *Wolbachia*, enabling efficient vertical transmission in *Trichogramma* wasps. Fluorescence *in situ* hybridization (FISH) was used to detect dynamic *Wolbachia* signals during early embryogenesis (30–120 min) in *T. dendrolimi* and *T. pretiosum*. Absolute quantitative polymerase chain reaction (AQ-PCR) and FISH were employed to clarify the loading and distribution dynamics of *Wolbachia* from the embryonic to adult stages of *T. dendrolimi* and *T. pretiosum*. The results of the present study provided a detailed description of *Wolbachia* dynamics during early embryogenesis and throughout different developmental stages of the host. These findings contribute to our understanding of how *Wolbachia* tropism enhances its vertical transmission across generations of *Trichogramma* wasps.

## Materials and methods

### Insects

*Wolbachia*-infected thelytokous isofemale *Trichogramma dendrolimi* and *T. pretiosum* lines were reared on eggs of the rice moth *Corcyra cephalonica* [Stainton] (Lepidoptera: Pyralidae) at 25°C ± 1°C and 75% ± 5% RH and under a 16-h/8-h light/dark cycle. The *C. cephalonica* host was reared on a semi-artificial diet.

### *Wolbachia* detection in *Trichogramma* by fluorescence *in situ* hybridization

FISH was conducted to detect *Wolbachia* signals in the early embryo (30, 50, 60, 70, 90, and 120 min) and at various developmental stages (24 h (late embryo), 48 h (larva), 72 h (larva), 120 h (pre-pupa), 168 h (pupa), and 240 h (adult)) of *T. dendrolimi* and *T. pretiosum* according to previously described procedures (Zhou et al., 2022a; Zhou et al., 2023). An egg card containing ~300 host eggs was introduced into a Durham’s tube (30 mm in length × 6 mm in diameter) and could be parasitized by ~500 *T. dendrolimi* or *T. pretiosum* females within 5 min. The parasitized eggs were then individually excised from the egg card at various times and dissected under a stereomicroscope (SV6; Carl Zeiss AG, Oberkochen, Germany) within 5 min. *Trichogramma* sample size uniformity was established and maintained by fixing the samples in Carnoy’s solution (1:3 (v/v) glacial acetic acid: methanol) for 48 h, rinsing them thrice in 50% (v/v) ethanol for 300 s each time, and treating them with destainer (6% (v/v) ethanol dissolved in perhydrol) for 48 h. The *Trichogramma* samples were then subjected twice to phosphate-buffered saline (PBS) for 300 s each time and transferred to a hybridization oven (HybriLinker HL-2000, UVP, USA) at 45 °C for 45 min. *Wolbachia* signals were indicated by two 5′-rhodamine-labeled *Wolbachia* probes (F: 5′-AATCCGGCCGAACCGACCC-3′ and R: 5′-CTTCTGTGAGTACCGTCATTATC-3′) targeting *Wolbachia* 16S rRNA. The *Wolbachia* distributions in the *Trichogramma* samples were photographed under a confocal laser scanning microscope (CLSM; Olympus FV3000, Monolith, Tokyo, Japan). The *Wolbachia* densities in the *Trichogramma* samples were quantified using the area of each *Wolbachia* signal weighted by the area of each *Trichogramma* sample *via* log transformation and with ImageJ software Version 1.53t (National Institutes of Health (NIH), Bethesda, MD, USA). To delineate asymmetric *Wolbachia* distributions in the *Trichogramma* samples, the early embryos were divided into anterior and posterior parts using the intermediate vertical boundaries of the lines from the anterior to posterior poles of the embryos. The symmetric ratios (SR) of the *Wolbachia* signals were calculated based on the odd ratios of the *Wolbachia* densities in the anterior and posterior parts of the embryos. The rates of the initial embryonic divisions were estimated based on the number of nuclei and the mitotic frequencies at various developmental times.

### *Wolbachia* titer quantification by AQ-PCR

*Trichogramma* progeny were dissected and isolated from their host eggs to evaluate the *Wolbachia* titers in *T. dendrolimi* and *T. pretiosum* at 48 h (larva), 72 h (larva), 120 h (pre-pupa), 168 h (pupa), and 240 h (adult) after parasitization. As *Trichogramma* embryos are very small, those that developed at ≤ 24 h were not subjected to AQ-PCR. In total, 50 progeny served as a sample replicate. The Chelex-100 method was used to extract total DNA from the *Trichogramma* samples. The *Wolbachia* titers in the *Trichogramma* samples were measured by AQ-PCR and a specific primer pair (F: 5′-ATGATGTAGCCCCAGAAAT-3′; R: 5′-CACCAAAAGTGTTGTAAAGAA-3′) that was designed according to the *Wolbachia wsp* sequences (GenBank Accession No. MG914000) (Zhou et al., 2019). The AQ-PCR method used here was previously described by Zhou et al. (2019).

### Data analysis

A structural equation model (SEM) was used to enumerate the nuclei and determine the mitotic frequency directly or indirectly influenced by the developmental stage, *Wolbachia* density, and SR (Grace, 2007). The SEM was simplified according to the lowest Akaike information criterion (AIC) value. The model was not rejected when the *χ* ^2^ test *p* > 0.05, the standardized root mean square residual (SRMR) < 0.1, and the comparative fit index (CFI) > 0.90. The standardized coefficient was used to estimate the correlation between variable pairs by weighting the units and the changes in the different variables (Abrams, 2007; Grace, 2007). A one-way analysis of variance (ANOVA) was used to analyze the influences of the developmental stage on the *Wolbachia* density and the SR. Tukey’s test was applied for *post hoc* comparisons. All analyses were conducted in R software version 4.2.0 (R Development Core Team, 2021).

## Results

### *Wolbachia* dynamics at the early *Trichogramma* embryo stage

**Figure 1:**
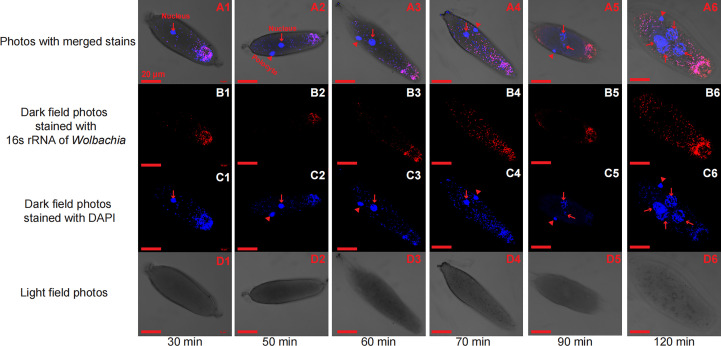
Early *T. dendrolimi* embryos stained with *Wolbachia* 16S rRNA (red) and DAPI (blue) probes at 30, 50, 60, 70, 90, and 120 min. A1, A2, A3, A4, A5, and A6 are light field photos stained with 16S rRNA of *Wolbachia* and DAPI signals. B1, B2, B3, B4, B5, and B6 are dark field photos stained with 16S rRNA of *Wolbachia*. C1, C2, C3, C4, C5, and C6 are dark field photos stained with DAPI signals. D1, D2, D3, D4, D5, and D6 are light field photos.

**Figure 2:**
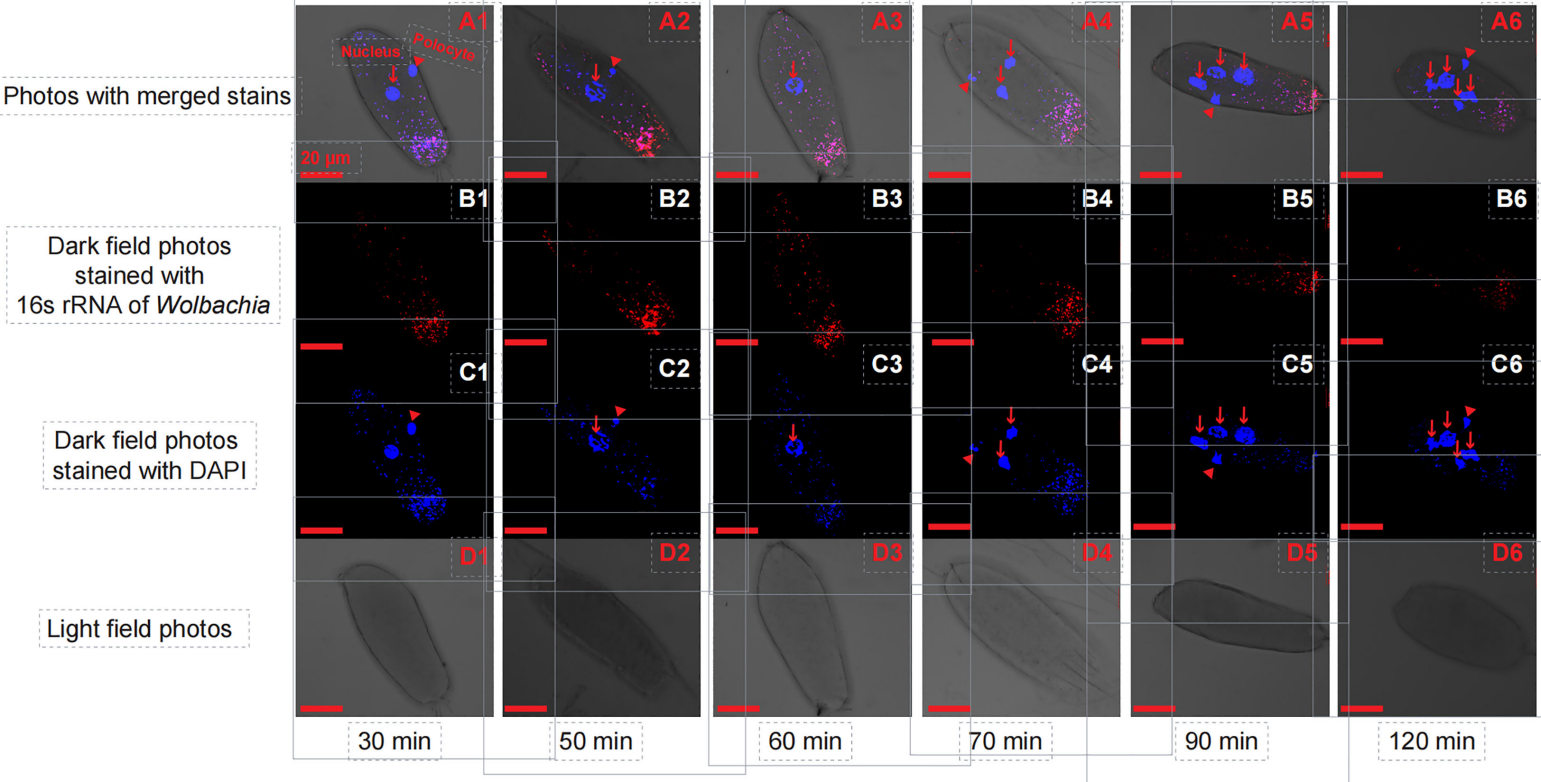
Early *T. pretiosum* embryos stained with *Wolbachia* 16S rRNA (red) and DAPI (blue) probes at 30, 50, 60, 70, 90, and 120 min. A1, A2, A3, A4, A5, and A6 are light field photos stained with 16S rRNA of *Wolbachia* and DAPI signals. B1, B2, B3, B4, B5, and B6 are dark field photos stained with 16S rRNA of *Wolbachia*. C1, C2, C3, C4, C5, and C6 are dark field photos stained with DAPI signals. D1, D2, D3, D4, D5, and D6 are light field photos.

In the initial divisions of both the *T. dendrolimi* and *T. pretiosum* embryos, we detected more *Wolbachia* signals at the posterior than the anterior part. The SR values for the *T. dendrolimi* embryos were 0.070 ± 0.013 (30 min), 0.13 ± 0.038 (50 min), 0.12 ± 0.027 (60 min), 0.30 ± 0.047 (70 min), 0.17 ± 0.026 (90 min), and 0.31 ± 0.030 (120 min). The SR values for the *T. pretiosum* embryos were 0.16 ± 0.021 (30 min), 0.16 ± 0.022 (50 min), 0.13 ± 0.025 (60 min), 0.12 ± 0.031 (70 min), 0.16 ± 0.028 (90 min), and 0.19 ± 0.035 (120 min) (**Figures 1**, **2**).

**Figure 3:**
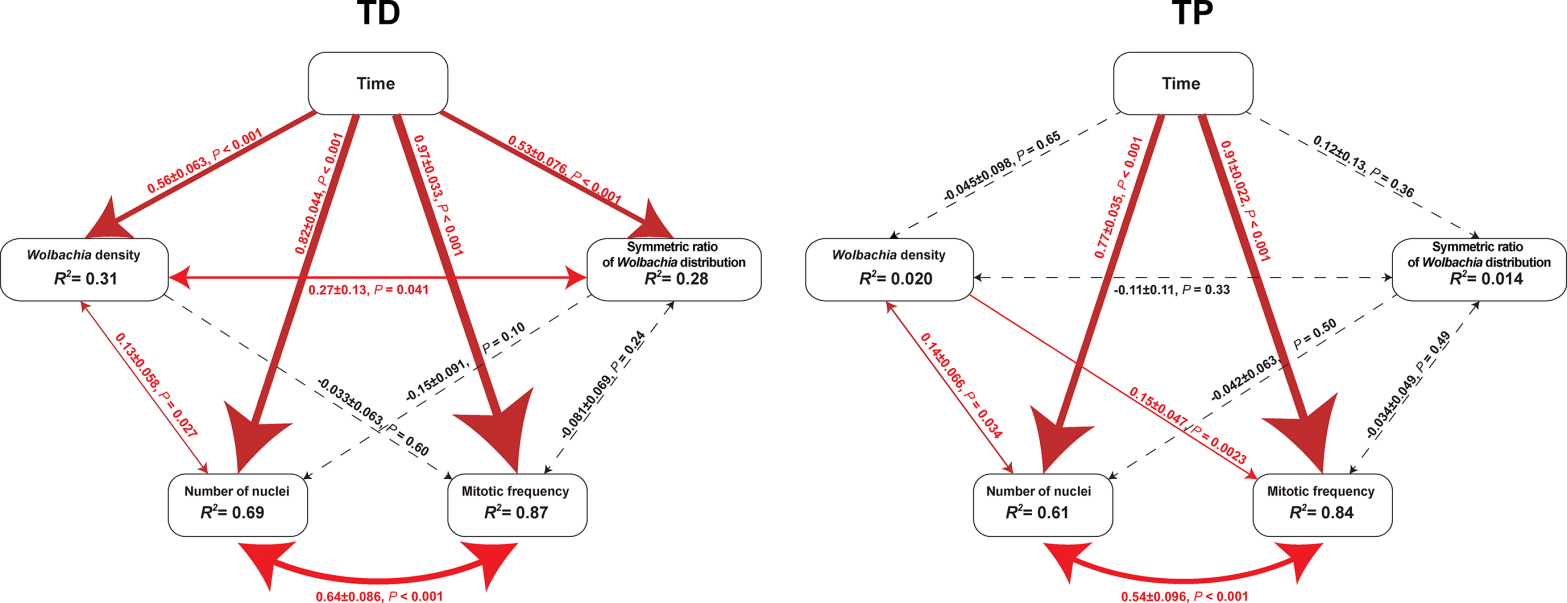
Diagrams of SEM paths of early embryogenesis in *T. dendrolimi* (TD) and *T. pretiosum* (TP). Red solid arrows indicate a significant correlation between the two variables. Black dashed arrows indicate a nonsignificant correlation between the two variables. The values alongside the lines indicate standardized coefficients and *p*-values for variable pairs.

**Figure 4:**
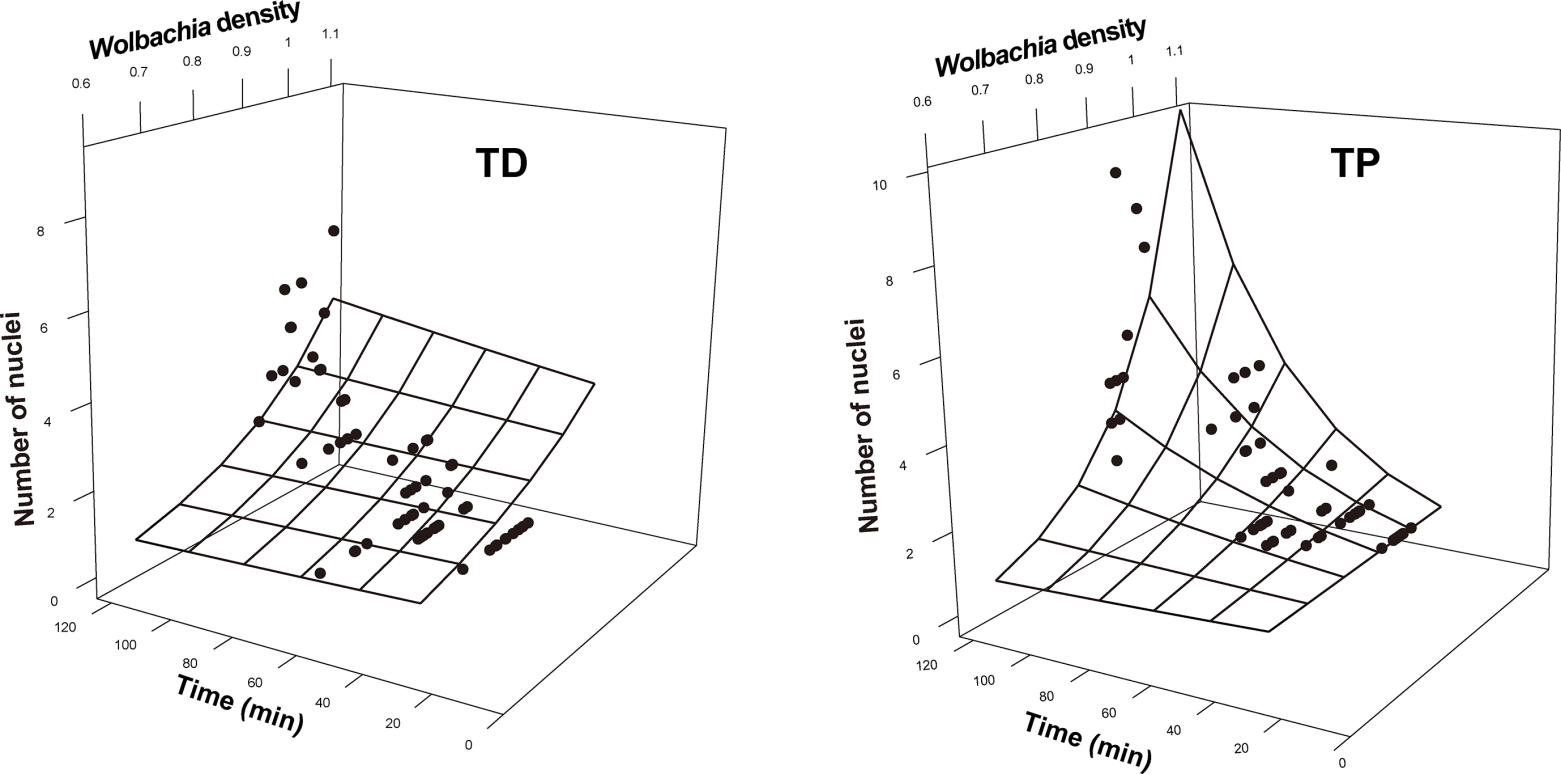
Number of nuclei as a function of developmental time and *Wolbachia* density in the early embryonic stages of *T. dendrolimi* (TD) and *T. pretiosum* (TP). The surface was estimated by log-linear models.

**Figure 5:**
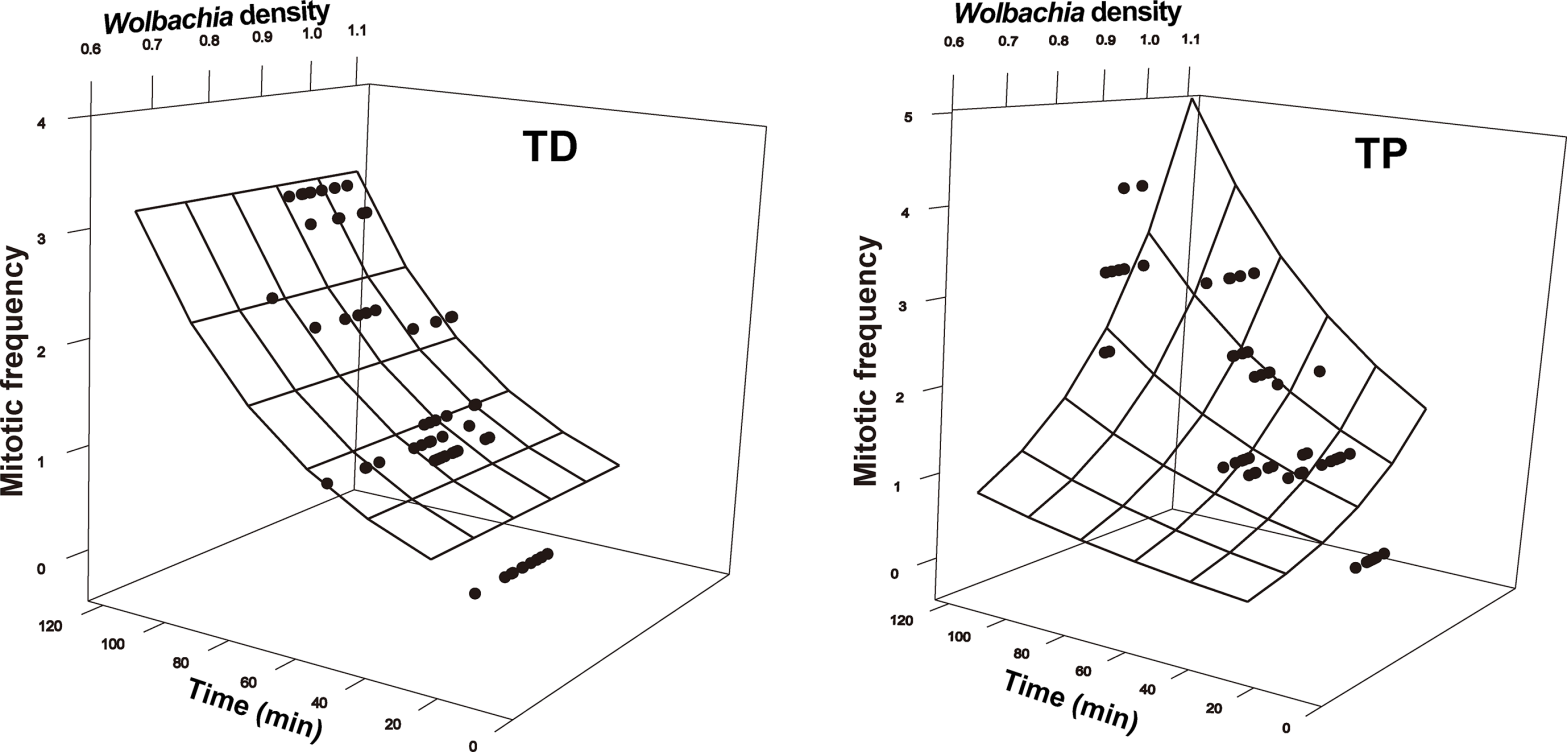
Mitotic frequency as a function of developmental time and *Wolbachia* density in the early embryonic stages of *T. dendrolimi* (TD) and *T. pretiosum* (TP). The surface was estimated by log-linear models.

The SEM for the *T. dendrolimi* embryos was not rejected after one path was excluded (*χ* ^2 =^ 0.28, *df* = 1, *p* = 0.60, CFI = 1.00, SRMR = 0.007). The *Wolbachia* density, SR, number of nuclei, and mitotic frequency increased with the stage of early embryonic development. The number of nuclei also increased with *Wolbachia* density, and the latter was positively correlated with the SR. The number of nuclei was positively correlated with the mitotic frequency (**Figures 3**–**5**).

The SEM for the *T. pretiosum* embryos was not rejected after two paths were excluded (*χ* ^2 =^ 1.35, *df* = 2, *p* = 0.50, CFI = 1.00, SRMR < 0.001). Early embryonic developmental time had no apparent effect on the *Wolbachia* density or the SR. The number of nuclei and the mitotic frequency increased with early embryonic development. Both the number of nuclei and the mitotic frequency increased with the *Wolbachia* density. The number of nuclei was positively correlated with the mitotic frequency. The correlation between the *Wolbachia* density and the SR was not significant (**Figures 3**–**5**).

In both *T. dendrolimi* and *T. pretiosum* embryos, we observed a higher number of *Wolbachia* signals at the posterior part compared to the anterior part. The number of nuclei and the mitotic frequency increased with the developmental time of the early embryo, irrespective of *Trichogramma* species. There was a positive correlation between *Wolbachia* density and the number of nuclei in both *T. dendrolimi* and *T. pretiosum* embryos. Additionally, the *Wolbachia* density, SR, and mitotic frequency increased with the developmental time of *T. dendrolimi* embryos, whereas this was not observed in *T. pretiosum* embryos.

### *Wolbachia* dynamics at different *Trichogramma* developmental stages

**Figure 6:**
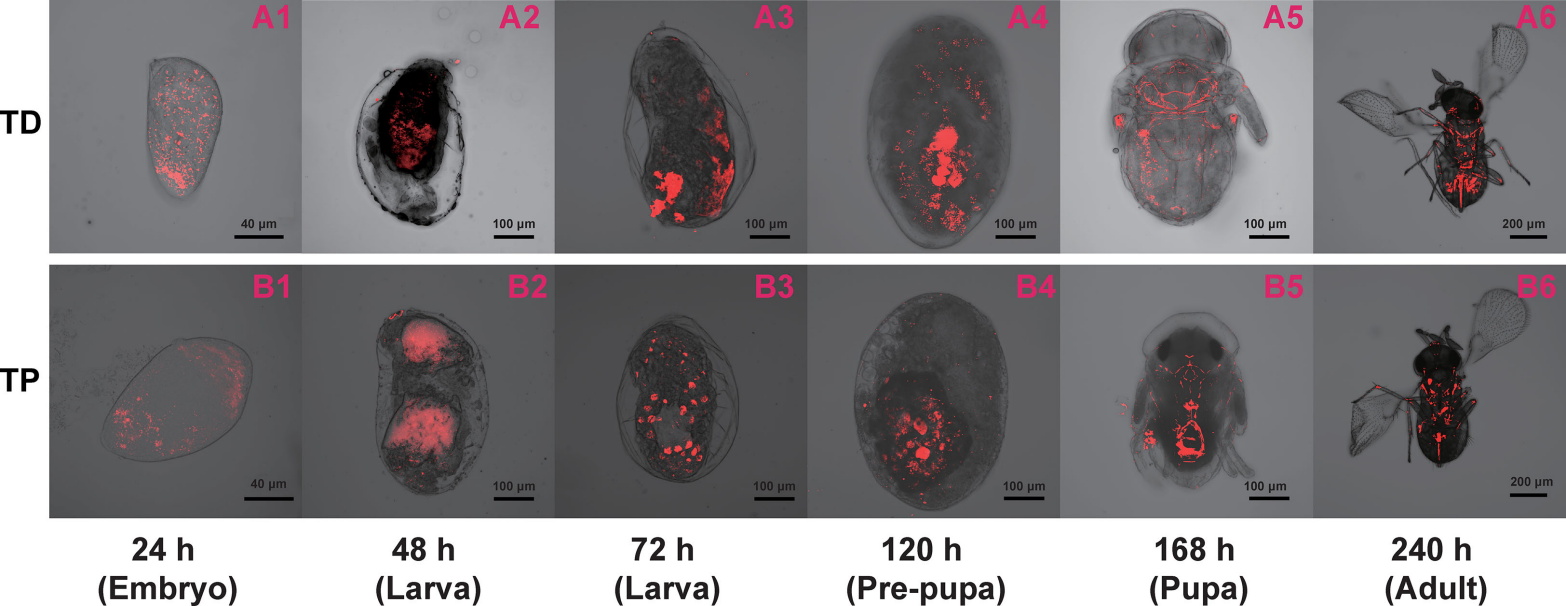
Immature *T. dendrolimi* (TD) and *T. pretiosum* (TP) offspring stained with *Wolbachia* 16S rRNA probes. A1, A2, A3, A4, A5, and A6 are *T. dendrolimi* offspring at 24, 48, 72, 120, 168, and 240 h, respectively. B1, B2, B3, B4, B5, and B6 are *T. pretiosum* offspring at 24, 48, 72, 120, 168, and 240 h, respectively.

**Figure 7:**
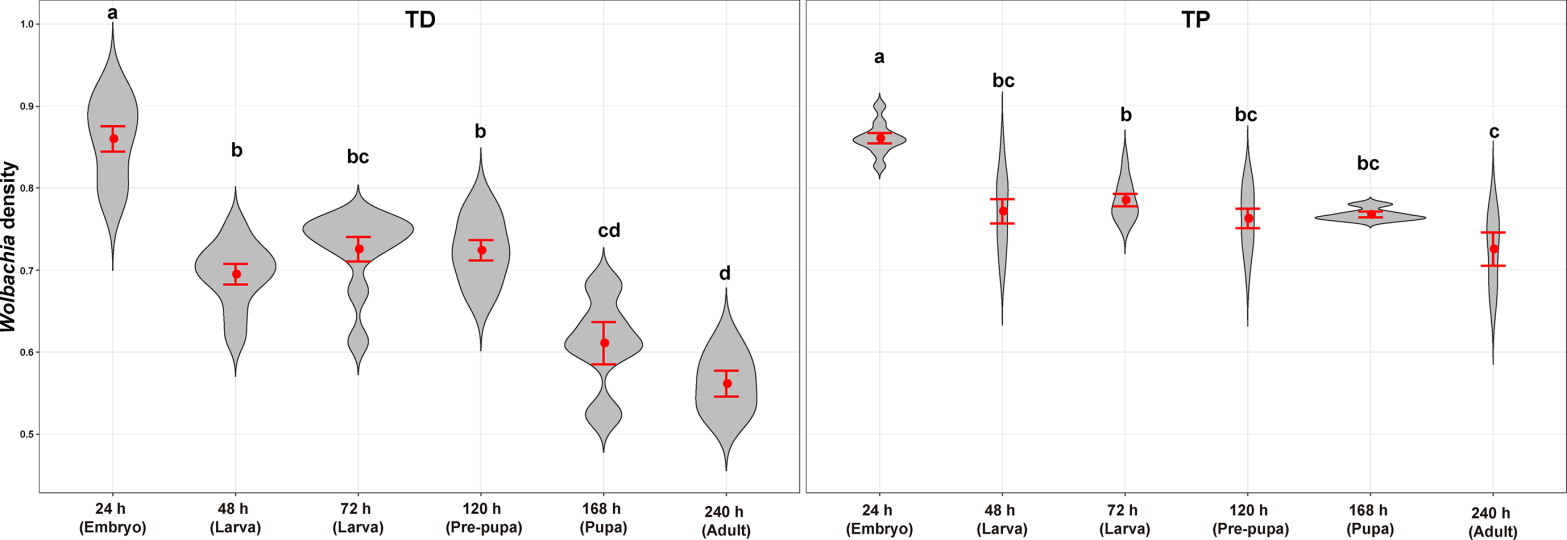
Symmetric ratios of the *Wolbachia* distribution of *T. dendrolimi* (TD) and *T. pretiosum* (TP) at different developmental stages. Red points indicate means. Error bars indicate 95% confidential intervals (CI). The red violin area indicates data distribution.

**Figure 8:**
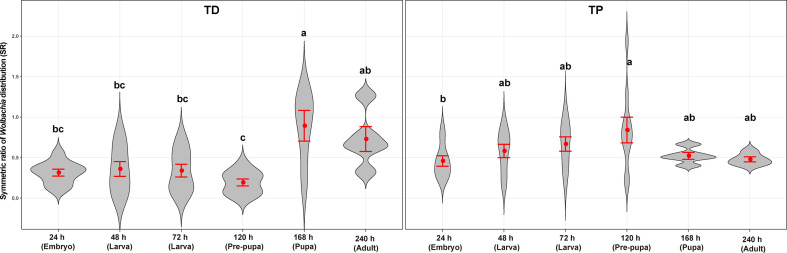
*Wolbachia* density of *T. dendrolimi* (TD) and *T. pretiosum* (TP) at different developmental stages. Red points indicate means. Error bars indicate 95% CI. The red violin area indicates data distribution.

The average SR values for the *T. dendrolimi* (0.19–0.89) and the *T. pretiosum* (0.46–0.84) offspring were < 1 at all stages (**Figures 6**, **7**). The average SR value for the *T. dendrolimi* offspring was highest at 168 h (0.89 ± 0.19) and significantly higher than those at 24 h (0.31 ± 0.042; *z* = 2.14, *p* = 0.032), 48 h (0.36 ± 0.091; *z* = 2.12, *p* = 0.033), 72 h (0.34 ± 0.079; *z* = 2.15, *p* = 0.031), and 120 h (0.19 ± 0.043; *z* = 3.18, *p* = 0.0015), but not significantly higher than that at 240 h (0.73 ± 0.15; *z* = 2.17, *p* = 0.86). For the *T. dendrolimi* offspring, the SR at 24, 48, 72, and 120 h did not significantly differ. For the *T. pretiosum* offspring, the average SR value was highest at 120 h (0.84 ± 0.16) and significantly higher than that at 24 h (0.46 ± 0.065; *z* = 2.45, *p* = 0.014). However, the SR at 48 h (0.58 ± 0.082), 72 h (0.67 ± 0.088), 120 h, 168 h (0.52 ± 0.043), and 240 h (0.48 ± 0.031) did not significantly differ (**Figure 7**). For the *T. dendrolimi* offspring, the average *Wolbachia* density was highest at 24 h (0.86 ± 0.016) and significantly higher than those at 48 h (0.69 ± 0.013; *z* = 3.82, *p* < 0.001), 72 h (0.73 ± 0.015; *z* = 2.62, *p* = 0.0087), 120 h (0.72 ± 0.012; *z* = 2.90, *p* = 0.0037), 168 h (0.61 ± 0.026; *z* = 4.65, *p* < 0.001), and 240 h (0.56 ± 0.016; *z* = 5.15, *p* < 0.001). The average *Wolbachia* density of the *T. dendrolimi* offspring was lowest at 240 h, significantly lower than those at 24 h, 48 h (*z* = 2.03, *p* = 0.042), 72 h (*z* = 2.50, *p* = 0.012), and 120 h (*z* = 2.28, *p* = 0.023), and not significantly lower than that at 168 h (*z* = 0.43, *p* = 0.66). The average *Wolbachia* density of the *T. pretiosum* offspring was highest at 24 h (0.86 ± 0.0062) and significantly higher than those at 48 h (0.77 ± 0.015; *z* = 3.62, *p* < 0.001), 72 h (0.79 ± 0.0076; *z* = 2.88, *p* = 0.0039), 120 h (0.76 ± 0.012; *z* = 3.93, *p* < 0.001), 168 h (0.77 ± 0.0035; *z* = 3.18, *p* = 0.0015), and 240 h (0.73 ± 0.020; *z* = 4.46, *p* < 0.001) (**Figure 8**).

**Figure 9:**
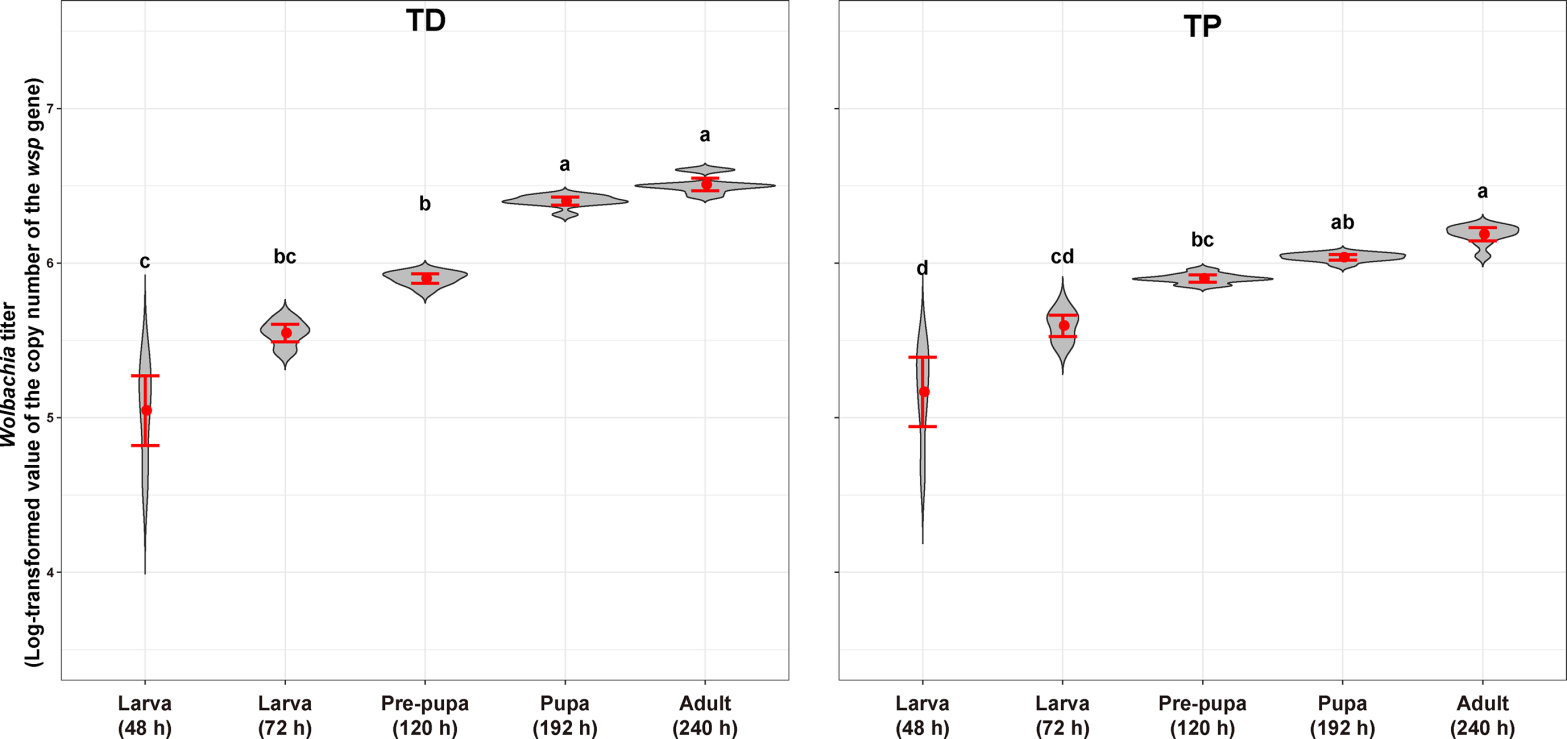
*Wolbachia* titer of *T. dendrolimi* (TD) and *T. pretiosum* (TP) at different developmental stages. Red points indicate means. Error bars indicate 95% CI. The red violin area indicates data distribution.

The *Wolbachia* titers in the *T. dendrolimi* and *T. pretiosum* offspring were evaluated by AQ-PCR. The average *Wolbachia* titer in the *T. dendrolimi* offspring was lowest at 48 h (5.05 ± 0.10), significantly lower than those at 120 h (5.90 ± 0.014; *z* = 3.07, *p* = 0.0035), 168 h (6.40 ± 0.012; *z* = 4.64, *p* < 0.001), and 240 h (6.51 ± 0.018; *z* = 6.10, *p* < 0.001), and not significantly lower than that at 72 h (5.55 ± 0.025; *z* = 1.53, *p* = 0.13). The *Wolbachia* titers in the *T. dendrolimi* offspring at 168 and 240 h were significantly higher than those at 24 and 72 h (*z* = 3.11, *p* = 0.0019; *z* = 4.56, *p* < 0.001) and 120 h (*z* = 2.10, *p* = 0.035; *z* = 3.03, *p* = 0.0024). The average *Wolbachia* titers in the *T. pretiosum* offspring were lowest at 48 h (5.17 ± 0.099), significantly lower than those at 120 h (5.90 ± 0.010; *z* = 2.98, *p* = 0.0029), 168 h (6.04 ± 0.0079; *z* = 4.67, *p* < 0.001), and 240 h (6.19 ± 0.019; *z* = 5.97, *p* < 0.001), and not significantly lower than that at 72 h (5.59 ± 0.031; *z* = 1.35, *p* = 0.18). The average *Wolbachia* titers were highest in the *T. pretiosum* offspring at 240 h, significantly higher than those at 48 and 72 h (*z* = 4.63, *p* < 0.001) and 120 h (*z* = 3.00, *p* = 0.0027), and not significantly lower than that at 168 h (*z* = 1.40, *p* = 0.16) (**Figure 9**).

The *Wolbachia* density and the SR were significantly influenced by the developmental stage in both *T. dendrolimi* and *T. pretiosum*. We observed a higher number of *Wolbachia* signals in the posterior part of each embryo compared to the anterior part. Regardless of *Trichogramma* species, *Wolbachia* density decreased with developmental time, while *Wolbachia* titers increased with developmental time.

## Discussion

*Wolbachia* presented efficient vertical transmission across host generations, as evidenced by the nearly 100% female offspring deposited by thelytokous *Trichogramma* females, all of which were infected by *Wolbachia*. We found that *Wolbachia* were concentrated in the posterior part of each embryo throughout the early stages of *T. dendrolimi* and *T. pretiosum* embryogenesis. Though the SR values varied with the *Trichogramma* wasp stage, the *Wolbachia* were nonetheless more concentrated in the posterior than the anterior regions of each embryo. These results provide evidence that the posterior concentration of *Wolbachia* during embryogenesis dynamically shapes tissue tropism during the postembryonic stages in two *Trichogramma* species, *T. dendrolimi* and *T. pretiosum*.

The distribution of *Wolbachia* in early embryos has been investigated in *Nasonia* and *Trichogramma* wasps, both parasitic wasps. As mentioned in the introduction, different patterns of *Wolbachia* distribution have been observed in these studies. Though *Wolbachia* exhibit posterior concentration in both *T. dendrolimi* and *T. pretiosum* embryos. The *Wolbachia* density, SR, and mitotic frequency increased with the developmental time of *T. dendrolimi* embryos, whereas this was not observed in *T. pretiosum* embryos. Similar to our findings, the *Wolbachia* wVitA strain in *N. giraulti* embryos localizes to the posterior end at a high density and then spreads toward the anterior end. Conversely, in *N. vitripennis* embryos, the *Wolbachia* wVitA strain localizes to the posterior end at a low density (Chafee et al., 2011; Funkhouser-Jones et al., 2018). In *Trichogramma* wasps, the posterior concentration of *Wolbachia* was also reported in *T. cordubensis* embryos, but not in *T. dendrolimi* (Pintureau et al., 2000). It is noteworthy that previous studies involving *Nasonia* or *Trichogramma* wasps have only described *Wolbachia* distribution at a single time point during embryogenesis, with one or several replicates. The process of *Wolbachia* tropism in postembryonic stages has not been described in parasitic wasps. The concentration of *Wolbachia* at the posterior pole during early embryogenesis is crucial for ensuring efficient vertical transmission. Notably, the distribution of embryonic *Wolbachia* varies widely among host arthropod species, including parasitoid species. The differences in *Wolbachia* localization patterns among insect host systems can be attributed to two factors. First, different *Wolbachia* strains may exhibit distribution patterns. For example, the tissue-specific distributions of *Wolbachia* were determined by its asymmetric localization and segregation during the initial mitotic divisions in early *Drosophila* embryos (Albertson et al., 2009). The *Wolbachia wPau* and *wWil* strains concentrated in the primordial germ cells of *D. paulistorum* and *D. willistoni* embryos, shaping the concentration of *Wolbachia* in the germ line. However, the broad distribution of *Wolbachia wMel* and *wWil* strains in *D. melanogaster* and *D. simulans* embryos results in systemic infections in different tissues of these species (Strunov et al., 2022). Second, the localization patterns of *Wolbachia* may vary among the embryos of different host species. For example, *Wolbachia* were concentrated in the anterior part of the embryos of the mosquito *Aedes polynesiensis* (Wright and Barr, 1981; Boyle et al., 1993) and the small brown planthopper *Laodelphax striatellus* (Guo et al., 2019), whereas a posterior *Wolbachia* concentration has been reported in embryos of the aphelinid *Aphytis* (Zchori-Fein et al., 1998). As mentioned earlier, the *Wolbachia wVitA* strain localizes to the posterior end at low density in the *N. vitripennis* embryo, but is concentrated in the posterior end at high density and then spread toward the anterior end in the *N. giraulti* wasp embryo (Chafee et al., 2011; Funkhouser-Jones et al., 2018). The foregoing results suggest that the *Wolbachia* localization patterns in the host embryo vary with the host background and *Wolbachia* strain. Phylogenetic studies have shown that 75% of *Wolbachia* strains from *Trichogramma* species belong to the *Sib* group clade in supergroup B (Almeida and Stouthamer, 2018). Our previous study confirmed that the *Wolbachia* strains from *T. dendrolimi* and *T. pretiosum* belong to the *Sib* group. Therefore, the present results suggest that the posterior concentration of *Wolbachia* during embryogenesis may be common in *Trichogramma* wasps infected by *Wolbachia* strains in the Sib group.

We found that *Wolbachia* density increased with the number of nuclei and the frequency of the initial mitotic divisions during early embryogenesis. According to the AQ-PCR, the total *Wolbachia* titer increased with *T. dendrolimi* and *T. pretiosum* offspring development. Relative to body size, however, *Wolbachia* density was significantly lower at the adult and pupal stages than in the embryo of *T. dendrolimi* and *T. pretiosum*. Similarly, the *Wolbachia* titers were high in the early embryos of *Drosophila sturtevanti*, *D. paulistorum*, and *D. willistoni* but low in late embryogenesis. For *D. septentriosaltans* and *D. simulans*, however, the *Wolbachia* titers were high during embryogenesis but relatively lower at the later developmental stages (Strunov et al., 2022). The observed reduction in *Wolbachia* density at the later stages of *Trichogramma* development may be explained by two factors. First, *Wolbachia* density may decrease later in host development because of the dilution effect of multiple cell divisions (Strunov et al., 2022). Second, *Wolbachia* activates host immune responses that could modify the distribution of the endosymbionts during the later developmental stages of the host. For example, the restricted *Wolbachia* infection pattern observed in the somatic tissues of *Drosophila* spp. is determined by selective autophagy involving interactions between *Wolbachia* and the endoplasmic reticulum (ER). ER stress could activate cell defense and immune responses (Celli & Tsolis, 2015; Strunov et al., 2022). Our study describes the dynamic pattern of *Wolbachia* distribution throughout the immature stages of *Trichogramma* hosts and serves as a fundamental reference for investigating the mechanisms underlying *Wolbachia* density and distribution dynamics. Thelytokous *Trichogramma* populations have the advantage of easier colonization in fields without the need for mating with males and potentially exhibit higher reproductive rates than their uninfected counterparts since all offspring are female (Zhou et al., 2019). The PI strength in *Trichogramma* wasps is strongly influenced by *Wolbachia* density in the germ line and eggs. Previous studies showed that weak PI strength is often associated with biotic (e.g., continuous oviposition) or abiotic (e.g., high temperature) factors that can affect the transmission rate of *Wolbachia* (Zhou et al., 2022a; Guo et al., 2023). The unstable PI strength of *Wolbachia*-infected *Trichogramma* can impact the quality of *Trichogramma* offspring and their efficacy in pest control. Our findings have practical implications for the future application of *Wolbachia*-infected *Trichogramma* in biological control programs.

Though *Wolbachia* were concentrated at the posterior part of the embryo throughout early embryogenesis, the SR values were significantly higher at the pupal stage of *T. dendrolimi* and the prepupal stage of *T. pretiosum* than at the embryonic stage. We detected *Wolbachia* signals in the anterior part of the embryo, the 48- and 72-h larvae, and the pre-pupae of *T. dendrolimi* and *T. pretiosum*. However, no *Wolbachia* signals were detected in the heads of pupal or adult *T. dendrolimi* and *T. pretiosum*. Our previous study revealed that *Wolbachia* infection caused memory loss, increased the incidence of superparasitism, and attenuated the host discrimination mechanism in *T. dendrolimi* (Zhou et al., 2022a). Though the *Wolbachia* titers in the heads of *Trichogramma* adults were low, the endosymbiont nonetheless caused behavioral changes in the wasps. The results of the present work suggest that *Wolbachia* colonization in the nervous tissues of immature *Trichogramma* progeny may impair central nervous system development and function in this insect host. However, the precise mechanisms by which *Wolbachia* infection induces behavioral changes in *Trichogramma* wasps remain to be elucidated.

The present study showed that posterior *Wolbachia* concentration during early *Trichogramma* embryogenesis dynamically shapes *Wolbachia* localization in this host wasp. In this way, *Wolbachia* can undergo efficient vertical transmission across generations and ensures that thelytokous *Trichogramma* females deposit only female *Wolbachia*-infected offspring. The present work clarified the localization pattern of *Wolbachia* during the embryogenesis and various developmental stages of *Trichogramma* spp. These findings may help understand the interactions between *Wolbachia* and other host insects and provide a fundamental reference for the application of *Wolbachia*-infected thelytokous *Trichogramma*.

## Data availability statement

The original contributions presented in the study are included in the article. Further inquiries can be directed to the corresponding authors.

## Author contributions

This study was designed by J-CZ, W-NC, HD, and L-SZ. Q-JD, DS, S-FN, and YW carried out the experiments. J-CZ, Q-JD, and DS also help to analyze the phenotype as well as the data. J-CZ, W-NC, and H-HZ also participated in the data analysis. J-CZ wrote the paper. All authors contributed to the article and approved the submitted version.
